# Normal Bone Microstructure and Density But Worse Physical Function in Older Women Treated with Selective Serotonin Reuptake Inhibitors, a Cross-Sectional Population-Based Study

**DOI:** 10.1007/s00223-018-0427-z

**Published:** 2018-05-05

**Authors:** Berit Larsson, Dan Mellström, Lisa Johansson, Anna G. Nilsson, Mattias Lorentzon, Daniel Sundh

**Affiliations:** 1 Geriatric Medicine, Department of Internal Medicine and Clinical Nutrition, Institute of Medicine, University of Gothenburg, Gothenburg, Sweden; 2 Geriatric Medicine, Sahlgrenska University Hospital, Building K, 6th floor, 431 80 Mölndal, Sweden; 3 Department of Orthopaedics, Sahlgrenska University Hospital, Gothenburg, Sweden

**Keywords:** SSRI, Physical function, Fall-related injury, Osteoporotic fracture, Bone microstructure

## Abstract

Depression in the elderly is today often treated with selective serotonin reuptake inhibitors (SSRIs) because of their favorable adverse effect profile. However, treatment with SSRIs is associated with increased risk of fractures. Whether this increased risk depends on reduced bone strength or increased fall risk due to reduced physical function is not certain. The aim was therefore to investigate if treatment with SSRIs is associated with impaired bone microstructure, bone density, or physical function in older women. From an ongoing population-based study, 1057 women (77.7 ± 1.5 years) were included. Validated questionnaires were used to assess information regarding medical history, medications, smoking, mental and physical health, and physical activity. Physical function was measured using clinically used tests: timed up and go, walking speed, grip strength, chair stand test, and one leg standing. Bone mineral density (BMD) was measured at the hip and spine with dual-energy X-ray absorptiometry (Hologic Discovery A). Bone geometry and microstructure were measured at the ultradistal and distal (14%) site of radius and tibia using high-resolution peripheral quantitative computed tomography (HR-pQCT; XtremeCT). Treatment with SSRIs was associated with higher BMD at the femoral neck, total hip, and lumbar spine, whereas no associations were found for any HR-pQCT-derived measurements. The use of SSRIs was associated with lower grip strength, walking speed, and fewer chair stand rises. These associations were valid also after adjustments for known risk factors for falls. Treatment with SSRIs was, independently of covariates, associated with worse physical function without any signs of inferior bone geometry and microstructure.

**Electronic supplementary material:**

The online version of this article (10.1007/s00223-018-0427-z) contains supplementary material, which is available to authorized users.

## Introduction

Bone fragility with fractures is today a growing public health concern, resulting in suffering and major cost for health care [1]. Today, the lifetime risk is 50% for women and 20% for men to suffer from an osteoporotic fracture [2] where hip fracture is the most severe. Of people with hip fracture, 80% survive the first year and only 30% regain their normal level of activity [3]. Fracture incidence is highly associated with low bone mineral density (BMD) [4] but other risk factors, such as falls, probably also play a large part in fracture risk. For individuals aged 65 years or older, 30% falls one or more times per year [5]. Few of the falls result in fracture, but 90% of all hip fractures occur after a fall [6]. The number of falls increases with age [7] and as the population continues to become older [8] an increase in fall-related injuries can be expected.

Selective serotonin reuptake inhibitors (SSRIs) are the most common medication for treatment of depression and are prescribed to 18% of the elderly women in Sweden [9]. Treatment with SSRIs is associated with an increased risk of fractures in older men and women [10, 11]. Whether this increased risk of fracture is driven by an effect on the bone tissue or general physical function, leading to falls, is not clear. SSRIs function by inhibition of the uptake of serotonin through interaction with the serotonin transporter [12]. This transporter has been found to be fully functional in osteoblasts, osteoclasts, and osteocytes, which could mediate an effect of SSRIs on bone metabolism. Treatment with SSRIs has been reported to be associated with reduced BMD as well as with increased bone loss [13, 14] in some but not all studies [15]. However, BMD does not provide information beyond bone mass and does not take bone microarchitecture into account.

Bone microstructure was earlier only possible to assess using invasive bone biopsies. With the introduction of high-resolution peripheral quantitative computed tomography (HR-pQCT), non-invasive images can be obtained at the radius and tibia [16]. Few studies have investigated the effect of SSRI treatment on bone microstructure, but higher levels of circulating serotonin have been shown to be associated with lower total and trabecular volumetric BMD at the femoral neck, assessed using QCT, as well as with fewer and thinner trabeculae at the radius measured using HR-pQCT [17].

Although the increased fracture risk for SSRI-treated patients may be mediated through bone metabolism, such an effect would be highly time-dependent. Studies have shown that treatment start with SSRIs results in an increased risk of hip fracture already within the first 6 weeks [18], indicating that a change in physical function and not altered bone phenotype may contribute to an elevated fracture risk. An increased risk of hip fracture could be secondary to an increased risk of falls, generated either by SSRI treatment or by the diseases themselves. Indeed, treatment with SSRIs has been reported to be associated with an increased risk of falls, independently of a diagnosis of depression [19].

The aim of the present population-based study was to investigate if SSRI use was associated with impaired bone microstructure, BMD or physical function in older women.

## Materials and Methods

### Subjects

The present study is a cross-sectional population-based study performed in the greater Gothenburg area. A total of 1057 women aged 75–80 were randomly selected and recruited via the Swedish national population register. An invitation letter was first sent to the participating women. They were later contacted by telephone and asked to participate. Those who accepted and had the ability to participate (were ambulant and able to follow instructions in Swedish) were invited to a visit at the Osteoporosis Clinic, Department of Geriatrics, Sahlgrenska University Hospital, Mölndal, Sweden. Prior to participation, all subjects signed an informed consent. The ethical review board at the University of Gothenburg has approved the study protocol.

### Anthropometrics and Physical Function Tests

Body height and weight were measured by standardized equipment. Two consecutive measurements of body height were performed and an average was calculated. If there was a difference of ≥ 5 mm, a third measurement was carried out, and the two most similar estimates were used. Several tests were used to evaluate physical function. Lower body strength [20] was determined using the 30-s chair stand test. It measures the maximum number of times the participant could rise up from a chair, in 30 s, with their arms crossed over their chest. A combined measure of mobility and balance, Timed Up and Go (TUG) [21] was performed. The participant was observed and timed while she rose from an arm chair, walked 3 m, turned, went back, and sat down again. One leg standing test [22] was used to measure balance. After the participants had a practice round, the test was performed twice for each leg. The best time for each leg was used to calculate an average for both legs. For measurement of walking speed, a 10-m walk test was used [23]. The participants were asked to walk 10 m twice at a self-chosen speed. The first 2 m was used for acceleration and the last two for deceleration. The middle 6 m was timed, and an average of the two trials was used to calculate a walking speed that was used in the analysis. Assessment of grip strength [24] was measured with a Saehan hydraulic hand dynamometer (model SH5001; Saehan Corporation, Masan, South Korea). The participants made two attempts with each hand, with the elbow held at a 90° angle and the lower arm resting on at flat surface. In this study, an average value for all four attempts was used.

### Questionnaires

All participants were asked to answer a standardized questionnaire concerning medical and fracture history, use of medication, current smoking, physical activity, occurrence of falls in the last 12 months, alcohol consumption, heredity of hip fracture, and calcium intake. Women defined as SSRI users in the current study had reported a daily use within the last 30 days. Daily calcium intake was assessed by combining the amount of calcium provided by supplements with food-derived calcium intake estimated by a validated questionnaire [25]. The participants were asked whether either of their parents had had a hip fracture and if they had experienced a fracture themselves, and if so, at what location and age. All fractures after the age of 50, except for head fractures, were considered. Physical activity during the last 7 days prior to the inclusion was estimated with the validated questionnaire Physical Activity Scale for the Elderly (PASE), which is a self-reported questionnaire that generates a total score of physical activity in individuals over 65 years by multiplying participation (yes/no) and the number of hours spent per week in different activities by given weights [26]. The SF-12 Health Survey was used to map the opinion of the participants self-rated health [27]. SF-12 tracks how the participant felt and how well they were able to perform usual activities and generates a component scale for physical health (PCS) as well as a measure of mental health reflecting common mental disorders such as depression (MCS).

### Dual-Energy X-Ray Absorptiometry

Assessment of aBMD and body composition was performed with dual-energy X-ray absorptiometry (DXA) using a Hologic Discovery A device (Hologic, Waltham, MA, USA). The BMD measurements were performed at the femoral neck, total hip, and lumbar spine (L1–L4). The amount of fat and lean body mass was estimated with a total body scan. Appendicular lean mass was assessed as lean mass/height^2^. For women in this age group, the coefficient of variations (CVs), at our facility, were 1.1% for fat mass, 0.6% for lean mass, 0.7% for lumbar spine aBMD, 0.8% for total hip aBMD, and 1.3% for femoral neck aBMD.

### High-resolution Peripheral Quantitative Computed Tomography

Using XtremeCT (Scanco Medical AG, Brüttisellen, Switzerland), an HR-pQCT equipment, bone microstructure and geometry was measured at the non-dominant arm (radius) and lower leg (tibia) of the corresponding side. All participants were measured using the standardized protocol recommended by the manufacturer, which has been described earlier [28]. With this protocol, the first image was obtained at 9.5 and 22.5 mm proximal to the reference line for radius and tibia, respectively (ultradistal site). To be able to better characterize cortical bone, a more proximal measurement was performed where the images were obtained at 14% of measured bone length from the end plate (distal site) [29]. Each measurement generated 110 slices, with a nominal isotropic resolution of 82 µm, which enabled a 3D construction of the bone. All images were processed [30] and generated the following variables: trabecular bone volume fraction (BV/TV, %), trabecular thickness (Tb.Th, mm), trabecular separation (Tb.Sp, mm), trabecular number (Tb.N, mm^−1^), cortical area (Ct.Ar, mm^2^), cortical thickness (Ct.Th, mm), and cortical volumetric bone mineral density (Ct.vBMD, mg/cm^3^). All generated variables were analyzed for the ultradistal section, where trabecular bone is more abundant, whereas only cortical variables were analyzed at the distal section. Every stack of images was given a level of quality ranging from 1 to 5, where one corresponds to perfect quality and five to suboptimal quality. Only images with a quality level between 1 and 3 were considered for analysis concerning bone geometry and microstructure variables. The number of measurements excluded due to poor quality related to motion artifacts was as follows: 38 for the ultradistal tibia images, 20 for the distal tibia images, 174 for the ultradistal radius images, and 82 for the distal radius images. In addition, 3 ultradistal tibia, 2 distal tibia, 13 ultradistal radius, and 9 distal radius images were excluded due to either presence of osteosynthesis material, deformed bone due to prior fracture, or inability to position the study subject.

The CVs were calculated using duplicate measurements in women between 75 and 80 years of age at the tibia (*n* = 6) and radius (*n* = 3). The CVs for bone variables generated at the tibia ultradistal site were as follows: 0.8% for BV/TV, 1.9% for Tb.N, 2.6% for Tb.Th, 2.1% for Tb.Sp, 0.2% for Ct.Th, 0.2% for Ct.Ar, and 0.2% for Ct.vBMD. At the distal tibia, the CVs were as follows: 0.4% for Ct.Ar, 0.3% for Ct.Th, and 0.2% for Ct.vBMD. At the ultradistal radius, the following CVs were obtained: 0.4% for BV/TV, 2.4% for Tb.N, 1.9% for Tb.Th, 2.4% for Tb.Sp, 1.2% for Ct.Th, 0.9% for Ct.Ar, and 0.4% for Ct.vBMD. At the distal radius, the CVs were as follows: 0.1% for Ct.Ar, 0.3% for Ct.Th, and 0.1% for Ct.vBMD.

### Cortical Evaluation

To further process all images, an Image Processing Language (IPL v5.08b), provided by the manufacturer (Scanco Medical AG), was used [31]. Contours were automatically placed at the periosteal and endosteal sides of the cortical bone, to separate cortical from trabecular bone and to delineate the bone from extra-osseal soft tissue. All contours were carefully inspected and were manually corrected if needed. Cortical porosity was obtained with the following equation: cortical pore volume/(cortical pore volume + cortical bone volume) [31, 32]. In the tibia, the CV for cortical porosity was 0.9% at the ultradistal site and 4.1% at the distal site. The CV for cortical porosity at the radius was 5.3% at the ultradistal site and 13.3% at the distal site. Cortical porosity analyses were only available on a subset of women of the included cohort and with numbers differing depending on analyses site (tibia ultradistal site: 64 SSRI-treated and 752 non-treated women; tibia distal site 64 and 761 women; radius ultradistal site: 57 and 644; radius distal site 62 and 712). In the subanalysis with matched controls, cortical porosity was available for the following number of women: tibia ultradistal 64 SSRI-treated and 267 without treatment; tibia distal 64 and 268, radius ultradistal 57 and 224; radius distal 62 and 243 women.

### Statistical Analyses

Differences between groups were investigated using independent samples t-test for continuous variables and χ^2^ and Fisher’s exact test for dichotomous variables. Linear regression analysis was used to investigate the association between use of SSRIs and bone variables adjusted for the following covariates: age, height, weight, MCS, PCS, oral glucocorticoid usage, rheumatoid arthritis, current smoking, heredity of hip fracture, previous fracture after the age of 50, and high alcohol consumption (more than three standard drinks per day). Linear regression analysis was also used to investigate the association between SSRI usage and physical function adjusted for the following covariates: age, height, weight, prior stroke, and MCS. In order to mitigate the possible effect of confounders on the associations between SSRI treatment and bone microstructure, a subanalysis comparing bone traits between SSRI-treated women and matched controls was performed. Four controls for every SSRI-treated woman were identified using R [RStudio Team (2016). RStudio: Integrated Development for R. RStudio, Inc., Boston, MA URL http://www.rstudio.com/] and the package MatchIt [33]. The matching procedure was performed based on age, height, weight, and prior stroke. Unless otherwise stated, values are presented as mean ± standard deviations for continuous variables and percentage together with number of participants for dichotomous variables. For linear regression models, a standardized beta value was presented. *p* values less than 0.05 were considered significant. SPSS Statistics Version 21 (IBM Corporation, Armonk, NY, USA) was used for all statistical analyses.

## Results

### Cohort Characteristics

From the 1057 women (77.7 ± 1.53 years), included in this population-based study, a total of 86 women were defined as treated with SSRIs and were compared against 971 non-treated controls (Table 1). Complete data were not available for all variables included in the analysis and information regarding missing data is therefore presented in a supplemental table (Table S1).

**Table 1:** Characteristics of older women with and without SSRI treatment

	SSRI No (*n* = 971)	SSRI Yes (*n* = 86)	*p*
Age (years)	77.7 ± 1.5	77.9 ± 1.6	0.38
Height (cm)	161.9 ± 5.81	162.5 ± 5.79	0.34
Weight (kg)	68.4 ± 12.0	69.7 ± 11.7	0.37
Appendicular lean mass index (kg/m^2^)	6.58 ± 0.89	6.51 ± 0.80	0.52
Fat mass (kg)	26.9 ± 7.49	27.4 ± 7.29	0.52
Age at menopause (years)	49.8 ± 4.85	50.2 ± 4.09	0.46
Physical activity score (PASE)	109 ± 52.9	92.9 ± 52.0	**0.01**
Calcium intake (mg/day)	704 ± 392	733 ± 411	0.53
MCS	54.8 ± 8.32	48.8 ± 11.3	< **0.001**
PCS	45.6 ± 10.8	42.0 ± 10.9	**0.003**
Fall accident last year, % (*n*)	30.2 (293)	39.5 (34)	0.07
Fracture, % (*n*)	4.8 (47)	3.5 (3)	0.57
Head injury, % (*n*)	6.7 (65)	10.5 (9)	0.19
Sprain, % (*n*)	2.8 (27)	3.5 (3)	0.71
Bruise, % (*n*)	12.9 (125)	24.4 (21)	**0.003**
Hyperthyroidism, % (*n*)	6.2 (60)	7.0 (6)	0.78
Hypothyroidism, % (*n*)	13.6 (131)	10.5 (9)	0.42
Diabetes, % (*n*)	9.2 (89)	11.6 (10)	0.46
Glucocorticoids p.o., % (*n*)	2.9 (28)	1.2 (1)	0.51^a^
Parkinson’s disease, % (*n*)	0.6 (6)	1.2 (1)	0.45^a^
Rheumatoid arthritis, % (*n*)	3.6 (35)	2.3 (2)	0.54
Prior stroke, % (*n*)	7.7 (75)	15.1 (13)	**0.02**
Current smoking, % (*n*)	5.3 (51)	5.8 (5)	0.82
Previous fracture, % (*n*)	37.5 (364)	37.2 (32)	0.96
Vertebral fracture, % (*n*)	4.8 (46)	5.9 (5)	0.64
Peripheral fracture, % (*n*)	24.3 (235)	25.9 (22)	0.75
Heredity of hip fracture, % (*n*)	14.7 (143)	18.6 (16)	0.34
High alcohol intake, % (*n*)	0.2 (2)	2.3 (2)	< **0.04**^a^
Chronic liver disease, % (*n*)	0.4 (4)	1.2 (1)	0.34^a^
Celiac disease, % (*n*)	1.2 (12)	1.2 (1)	1.00^a^

Statistically significant values (*p* < 0.05) are given in bold

An independent samples *t* test was used to compare differences in continuous variables between SSRI-users and non-users. Proportions for dichotomous variables were compared by chi-square test or Fisher’s exact test for small sample sizes. High alcohol intake was defined as more than three standard drinks per day

*SSRI* selective serotonin reuptake inhibitors, *MCS* mental health component scale, *PCS* physical health component scale. Previous fracture = fracture after the age of 50

^a^Fisher’s exact test

The women treated with SSRIs had lower mental and physical health compared to women without treatment. They were also less physically active than non-treated women according to PASE (Table 1). Among women with SSRI treatment, 15.1% had a previous stroke, compared to only 7.7% in those without SSRI (*p* = 0.02). No significant differences between the groups were found regarding age, height, weight, prior fracture, heredity of fracture, daily calcium intake, fall accident last year, oral glucocorticoid treatment, rheumatoid arthritis, appendicular lean mass index, diabetes, Parkinson’s disease, or smoking (Table 1). Even though fall accident the year prior to study inclusion did not differ, the SSRI-treated women experienced a higher rate of bruising after a fall meanwhile no difference was seen for fracture, sprain, or head injury (Table 1).

### Physical Function Indices

SSRI users had − 7.9% reduced walking speed, − 9.3% reduced grip strength, and − 11.5% fewer rises from a chair than women not taking SSRIs. No significant differences were seen for the one leg standing test or for the timed up and go test (Table 2).

**Table 2:** Physical function in older women with and without SSRI treatment

Function	SSRI No (*n* = 971)	SSRI Yes (*n* = 86)	*p* value	Standardized beta	Adjusted *p*-value
One leg standing (s)	14.9 ± 9.5	14.3 ± 8.8	0.58	− 0.006	0.85
Timed up and go (s)	8.7 ± 3.1	9.3 ± 2.9	0.06	0.02	0.46
Walking speed (m/s)	1.27 ± 0.24	1.17 ± 0.23	< **0.001**	− 0.08	**0.01**
Chair stand test (number/30 s)	11.3 ± 3.4	10.0 ± 3.0	**0.001**	− 0.07	**0.03**
Grip strength (kg)	12.9 ± 5.0	11.7 ± 4.8	**0.03**	− 0.06	**0.04**

Statistically significant values (*p* < 0.05) are given in bold

An independent samples *t* test was used to compare differences in indices of physical function between SSRI-users and non-users. Differences in these variables were also investigated in linear regression models, adjusted for age, weight, height, prior stroke, and mental health component scale (MCS). Results for the linear regressions are presented as standardized beta

*SSRI* selective serotonin reuptake inhibitors

After adjustment for age, height, weight, prior stroke, and MCS, associations were still apparent for the chair stand test (*p* = 0.03), grip strength (*p* = 0.04), and walking speed (*p* = 0.01) (Table 2).

### Areal BMD

SSRI users had higher BMD at the femoral neck (2.9%), total hip (4.8%), and lumbar spine (6.4%) than women without SSRI. These differences were also independent of covariates including age, height, weight, MCS, PCS, oral glucocorticoid treatment, rheumatoid arthritis, current smoking, high alcohol consumption, parental hip fracture, and previous fracture after the age of 50 (Table 3).

**Table 3:** Areal bone mineral density, bone geometry, and microstructure in older women with and without SSRI treatment

DXA	SSRI No	SSRI Yes	*p* value	Standardized beta	Adjusted *p* value
	(*n* = 970)	(*n* = 86)			
Femoral neck aBMD (g/cm^2^)	0.66 ± 0.1	0.68 ± 0.1	**0.02**	0.06	**0.02**
Total Hip aBMD (g/cm^2^)	0.79 ± 0.1	0.83 ± 0.1	**0.01**	0.07	**0.01**
Lumbar spine aBMD (g/cm^2^)	0.94 ± 0.2	1.00 ± 0.2	< **0.001**	0.08	< **0.01**

HR-pQCT					
Tibia ultradistal	(*n* = 935)	(*n* = 81)			
Trabecular bone volume fraction (%)	12.2 ± 3.0	12.6 ± 2.8	0.27	0.03	0.30
Trabecular thickness (mm)	0.07 ± 0.01	0.07 ± 0.01	0.70	0.004	0.90
Trabecular separation (mm)	0.52 ± 0.17	0.50 ± 0.14	0.22	− 0.03	0.40
Trabecular number (mm^−1^)	1.78 ± 0.36	1.84 ± 0.35	0.12	0.03	0.29
Cortical area (mm^2^)	78.4 ± 23.1	74.0 ± 21.3	0.10	− 0.04	0.21
Cortical volumetric BMD (mg/cm^3^)	740 ± 67.2	733 ± 62.6	0.39	− 0.01	0.80
Cortical thickness (mm)	0.75 ± 0.24	0.70 ± 0.22	0.06	− 0.04	0.22
Cortical porosity (%)	10.5 ± 2.86	10.6 ± 2.93	0.86	0.01	0.84

HR-pQCT					
Tibia distal (14% of bone length)	(*n* = 953)	(*n* = 82)			
Cortical area (mm^2^)	148 ± 24.2	148 ± 24.0	0.95	0.001	0.97
Cortical volumetric BMD (mg/cm^3^)	916 ± 42.5	919 ± 43.1	0.61	0.02	0.52
Cortical thickness (mm)	1.82 ± 0.32	1.80 ± 0.30	0.68	− 0.003	0.91
Cortical porosity (%)	4.65 ± 2.14	4.56 ± 2.07	0.74	− 0.02	0.57

HR-pQCT					
Radius ultradistal	(*n* = 800)	(*n* = 70)			
Trabecular bone volume fraction (%)	9.97 ± 3.44	10.6 ± 3.62	0.17	0.05	0.16
Trabecular thickness (mm)	0.06 ± 0.01	0.06 ± 0.01	0.49	0.03	0.42
Trabecular separation (mm)	0.59 ± 0.26	0.55 ± 0.20	0.25	− 0.04	0.24
Trabecular number (mm^−1^)	1.70 ± 0.43	1.78 ± 0.45	0.15	0.05	0.17
Cortical area (mm^2^)	37.8 ± 11.7	37.2 ± 9.77	0.69	− 0.02	0.62
Cortical volumetric BMD (mg/cm^3^)	772 ± 79.0	770 ± 69.3	0.88	0.01	0.78
Cortical thickness (mm)	0.55 ± 0.18	0.54 ± 0.16	0.82	− 0.003	0.93
Cortical porosity (%)	3.74 ± 1.68	3.70 ± 1.71	0.87	− 0.02	0.54

HR-pQCT					
Radius distal (14% of bone length)	(*n* = 889)	(*n* = 77)			
Cortical area (mm^2^)	59.2 ± 9.93	59.8 ± 10.1	0.61	0.01	0.78
Cortical volumetric BMD (mg/cm^3^)	1004 ± 39.0	1002 ± 35.7	0.62	0.001	0.98
Cortical thickness (mm)	1.32 ± 0.21	1.33 ± 0.19	0.68	0.02	0.61
Cortical porosity (%)	1.96 ± 1.64	1.98 ± 1.62	0.92	− 0.02	0.59

Statistically significant values (*p* < 0.05) are given in bold

An independent samples *t* test was used to compare differences in indices of bone variables between SSRI-users and non-users. Differences in these variables were also investigated in linear regression models, adjusted for age, height, weight, mental health (MCS), physical health (PCS), oral glucocorticoid treatment, rheumatoid arthritis, current smoking, high alcohol consumption (more than three standard drinks per day), parental hip fracture, and previous fractures. Results for the linear regressions are presented as standardized beta

*SSRI* selective serotonin reuptake inhibitor, *aBMD* areal bone mineral density, *HR-pQCT* high-resolution peripheral quantitative computed tomography, *Ultradistal* HR-pQCT measurements according to the manufacturer, *Distal* HR-pQCT measurements at 14% of tibia bone length

### Bone Geometry and Microarchitecture

No bone traits measured at the ultradistal or distal site of radius and tibia using the HR-pQCT differed between groups, neither before nor after adjustment for age, height, weight, MCS, PCS, oral glucocorticoid treatment, rheumatoid arthritis, smoking, alcohol consumption, heredity of hip fracture, and previous fracture after the age of 50 (Table 3).

### Matching Women Treated with SSRI Against Non-users

The 86 women treated with SSRI were matched (ratio 1:4) to 344 women without treatment. The SSRI-treated women were similar in most baseline characteristics except having lower physical activity the last seven days (PASE-score), physical function and mental health measured with SF-12 (PCS and MCS) (Table 4). Women treated with SSRI had worse physical function in regards to lower walking speed, fewer chair stands, and lower grip strength than women not treated with SSRI. No difference was seen for timed up and go or one leg standing (Table 5). After adjustment for MCS, associations were still apparent for the chair stand test (*p* = 0.03) and walking speed (*p* = 0.04) (Table 5). As for the primary analysis, the subanalysis examined associations between SSRI treatment, bone geometry and microstructure. Women treated with SSRI had higher aBMD measured at all three locations. In addition, no differences were seen for any of the geometry and microstructural measurements at either radius or tibia (Table 6).

**Table 4:** Characteristics of women with SSRI treatment and matched controls

	SSRI No (*n* = 344)	SSRI Yes (*n* = 86)	*p*
Age (years)	78.0 ± 1.6	77.9 ± 1.6	0.47
Height (cm)	162.8 ± 6.1	162.5 ± 5.8	0.78
Weight (kg)	70.0 ± 13.1	69.7 ± 11.7	0.84
Appendicular lean mass index (kg/m^2^)	6.64 ± 0.94	6.51 ± 0.80	0.25
Fat mass (kg)	27.6 ± 8.10	27.4 ± 7.29	0.81
Age at menopause (years)	50.1 ± 4.8	50.2 ± 4.1	0.80
Physical activity score (PASE)	105 ± 49.5	92.9 ± 52.0	**0.05**
Calcium intake (mg/day)	685 ± 369	733 ± 411	0.30
MCS	54.9 ± 8.3	48.8 ± 11.3	< **0.001**
PCS	44.7 ± 11.3	42.0 ± 10.9	**0.04**
Fall accident last year, % (*n*)	31.1 (107)	39.5 (34)	0.14
Hyperthyreodism, % (*n*)	5.5 (19)	7.0 (6)	0.61
Hypothyreodism, % (*n*)	14.3 (49)	10.5 (9)	0.35
Diabetes, % (*n*)	7.6% (26)	11.6% (10)	0.22
Glucocorticoids p.o., % (*n*)	2.6 (9)	1.2 (1)	0.69^a^
Parkinson’s disease, % (*n*)	1.2 (4)	1.2 (1)	1.00^a^
Rheumatoid arthritis, % (*n*)	4.9 (17)	2.3 (2)	0.29
Prior stroke, % (*n*)	12.8 (44)	15.1 (13)	0.57
Current smoking, % (*n*)	5.5 (19)	5.8 (5)	0.92
Previous fracture, % (*n*)	40.1 (138)	37.2 (32)	0.62
Heredity of hip fracture, % (*n*)	16.3 (56)	18.6 (16)	0.61
High alcohol intake, % (*n*)	0.3 (1)	2.3 (2)	0.10^a^
Chronic liver disease, % (*n*)	0.3 (1)	1.2 (1)	0.36^a^
Celiac disease, % (*n*)	1.2 (4)	1.2 (1)	1.00^a^

Statistically significant values (*p* < 0.05) are given in bold

An independent samples *t* test was used to compare differences in continuous variables between matched SSRI-users and non-users. Proportions for dichotomous variables were compared by chi-square test or Fisher’s exact test for small sample sizes. High alcohol intake was defined as more than three standard drinks per day

*SSRI* selective serotonin reuptake inhibitors, *MCS* mental health component scale, *PCS* physical health component scale. *Previous fracture* fracture after the age of 50

^a^Fisher’s exact test

**Table 5:** Physical function in women with SSRI treatment and matched controls

Function	SSRI No (*n* = 344)	SSRI Yes (*n* = 86)	*p-*value	Standardized beta	Adjusted *p*-value
One leg standing (s)	14.1 ± 9.3	14.3 ± 8.8	0.92	− 0.001	0.90
Timed up and go (s)	8.8 ± 3.6	9.3 ± 2.9	0.24	0.05	0.35
Walking speed (m/s)	1.25 ± 0.25	1.17 ± 0.23	**0.01**	− 0.10	**0.04**
Chair stand test (number/30 s)	10.9 ± 3.3	10.0 ± 3.0	**0.02**	− 0.11	**0.03**
Grip strength (kg)	12.9 ± 4.8	11.7 ± 4.8	**0.04**	− 0.09	0.08

Statistically significant values (*p* < 0.05) are given in bold

An independent samples *t* test was used to compare differences in indices of physical function between matched SSRI-users and non-users. Differences in these variables were also investigated in linear regression models adjusted for mental health (MCS). Results for the linear regressions are presented as standardized beta

*SSRI* selective serotonin reuptake inhibitors

**Table 6:** Areal bone mineral density, bone geometry, and microstructure in women with SSRI treatment and matched controls

DXA	SSRI No	SSRI Yes	*p*-value	Standardized beta	Adjusted *p*-value
	(*n* = 343)	(*n* = 86)			
Femoral neck aBMD (g/cm^2^)	0.65 ± 0.1	0.68 ± 0.1	**0.01**	0.13	**0.01**
Total Hip aBMD (g/cm^2^)	0.79 ± 0.1	0.83 ± 0.1	**0.01**	0.13	**0.01**
Lumbar spine aBMD (g/cm^2^)	0.94 ± 0.2	1.00 ± 0.2	**0.001**	0.15	**0.002**

HR-pQCT					
Tibia ultradistal	(*n* = 336)	(*n* = 81)			
Trabecular bone volume fraction (%)	12.1 ± 3.0	12.6 ± 2.8	0.17	0.07	0.16
Trabecular thickness (mm)	0.07 ± 0.01	0.07 ± 0.01	0.64	0.04	0.46
Trabecular separation (mm)	0.52 ± 0.18	0.50 ± 0.14	0.31	− 0.05	0.33
Trabecular number (mm^−1^)	1.80 ± 0.37	1.84 ± 0.35	0.33	0.04	0.43
Cortical area (mm^2^)	76.9 ± 23.7	74.0 ± 21.3	0.32	− 0.05	0.36
Cortical volumetric BMD (mg/cm^3^)	736 ± 71.0	733 ± 62.6	0.77	− 0.01	0.85
Cortical thickness (mm)	0.73 ± 0.25	0.70 ± 0.22	0.32	− 0.05	0.37
Cortical porosity (%)	10.4 ± 2.92	10.6 ± 2.93	0.59	0.04	0.52

HR-pQCT					
Tibia distal (14% of bone length)	(*n* = 341)	(*n* = 82)			
Cortical area (mm^2^)	148 ± 25.4	148 ± 24.0	0.99	− 0.0001	1.00
Cortical volumetric BMD (mg/cm^3^)	914 ± 43.8	919 ± 43.1	0.39	0.05	0.34
Cortical thickness (mm)	1.80 ± 0.33	1.80 ± 0.30	0.95	− 0.001	0.98
Cortical porosity (%)	4.71 ± 2.18	4.56 ± 2.07	0.62	− 0.04	0.49

HR-pQCT					
Radius ultradistal	(*n* = 278)	(*n* = 70)			
Trabecular bone volume fraction (%)	9.86 ± 3.33	10.6 ± 3.62	0.12	0.10	0.07
Trabecular thickness (mm)	0.06 ± 0.01	0.06 ± 0.01	0.56	0.05	0.37
Trabecular separation (mm)	0.59 ± 0.26	0.55 ± 0.21	0.22	− 0.07	0.19
Trabecular number (mm^−1^)	1.69 ± 0.43	1.78 ± 0.45	0.14	0.08	0.13
Cortical area (mm^2^)	37.4 ± 12.2	37.2 ± 9.77	0.93	− 0.01	0.81
Cortical volumetric BMD (mg/cm^3^)	764 ± 79.2	770 ± 69.3	0.51	0.03	0.53
Cortical thickness (mm)	0.54 ± 0.16	0.53 ± 0.19	0.68	0.01	0.82
Cortical porosity (%)	3.78 ± 1.59	3.70 ± 1.71	0.73	− 0.02	0.73

HR-pQCT					
Radius distal (14% of bone length)	(*n* = 311)	(*n* = 77)			
Cortical area (mm^2^)	59.8 ± 11.0	59.8 ± 10.1	0.98	0.002	0.97
Cortical volumetric BMD (mg/cm^3^)	1004 ± 38.9	1002 ± 35.7	0.65	− 0.002	0.97
Cortical thickness (mm)	1.32 ± 0.21	1.33 ± 0.19	0.76	0.02	0.69
Cortical porosity (%)	1.89 ± 1.38	1.98 ± 1.62	0.65	0.02	0.78

Statistically significant values (*p* < 0.05) are given in bold

An independent samples *t* test was used to compare differences in indices of bone variables between matched SSRI-users and non-users. Differences in these variables were also investigated in linear regression models, adjusted for mental health (MCS), physical health (PCS), oral glucocorticoid treatment, rheumatoid arthritis, current smoking, high alcohol consumption (more than three standard drinks per day), parental hip fracture, and previous fractures. Results for the linear regressions are presented as standardized beta

*SSRI* selective serotonin reuptake inhibitor, *aBMD* areal bone mineral density, *HR-pQCT* high-resolution peripheral quantitative computed tomography, *Ultradistal* HR-pQCT measurements according to the manufacturer, *Distal* HR-pQCT measurements at 14% of tibia bone length

## Discussion

In the present study, we report an association between SSRI treatment and higher aBMD at the femoral neck, total hip, and lumbar spine in older Swedish women independently of covariates, whereas no associations were detected with more detailed bone geometry and microstructural variables. However, SSRI treatment was associated with lower physical function, also after adjustment for covariates, indicating that the increased fracture risk in those treated with SSRIs [10, 11] could be explained by lower physical function leading to increased risk for falls.

SSRIs are considered as first-line therapy for the treatment of depressive symptoms among older adults because of their presumed favorable adverse effect profile [34]. However, serotonin transporters have recently been described in bone [14], raising the possibility that medications that block serotonin reuptake could affect bone metabolism. Evidence from previous cohort studies suggests that the use of antidepressants at therapeutic doses is associated with decreased BMD [13, 14] and increased fracture risk in both men and women [10, 11]. A prospective cohort study comprised 2722 community-dwelling older women (mean age, 78.5 years) demonstrated a 0.47% yearly loss in mean total hip BMD in non-users, compared with 0.82% in SSRI users (*p* < 0.001). Higher rates of bone loss were also observed at the trochanter and femoral neck for SSRI users [14]. In contrast, in our cohort of elderly women, use of SSRIs was associated with a higher aBMD at all clinical measurement sites. In addition, besides having better bone density, other important bone affecting variables included into the fracture assessment tool FRAX® (e.g., prior fracture, heredity of hip fracture, glucocorticoid treatment, and smoking) did not differ between the SSRI-treated subjects and controls, which indicate that the bone phenotype is similar between the two groups.

Areal BMD is a good measure of bone strength [35], but as a clinical tool the method has difficulties in predicting fractures in individuals with normal BMD [36]. A major reason for this could be the inability to separate trabecular from cortical bone. Whether treatment with SSRIs affects bone geometry and microstructure is not known. A study by Modder et al. showed that higher levels of circulating serotonin were associated with lower total and trabecular volumetric BMD at the femoral neck as well as with fewer and thinner trabeculae at the radius [17]. Our results do not indicate any effects on bone geometry or microstructure, measured at both radius and tibia, when comparing SSRI treatment with controls. However, it is difficult to compare these results since there might be large differences in function between serotonin levels located in the brain, affected by the treatment, and serotonin levels measured peripherally.

Increased fracture risk due to reduced BMD and deteriorated bone structure is a rather slow process. With a rather small difference in the yearly loss of BMD for SSRI-treated compared to non-treated subjects [14], the increased fracture risk would be apparent only after several years of treatment. Since SSRI treatment is associated with an increased fracture risk shortly after treatment start [18], it is unlikely that changes in BMD drives this association. If altered bone density or structure cannot explain the increased fracture risk, increased fall risk might. A higher fall risk could potentially be explained by lower physical function in the women treated with SSRIs compared to controls. This impaired physical function could not be explained by lower appendicular muscle mass, which indicate that muscle function rather than muscle mass is affected and is the main reason for the lower physical function that generates a higher falls risk.

Treatment with SSRIs is associated with an increased risk for falls [37], which might contribute to an increased fracture risk for SSRI-treated women. However, it is troublesome to separate the effect on fall risk of SSRI treatment from that of the depression itself, which is a diagnosis associated with increased fall risk [38]. In the present study, women treated with SSRIs reported a borderline significantly higher number of falls, possibly explained by worse physical function. This lower physical function could be derived from lower current physical activity and self-perceived physical health, which might be due to the depression, reflected by the lower mental health. Another condition heavily affecting physical function is stroke, and the proportion of prior stroke was higher for women treated with SSRIs. It is common that patients with a prior stroke develop depression [39], which is treated with SSRIs. Therefore, SSRIs could be a proxy for a worse physical function in reality explained by a prior stroke. However, SSRI treatment was associated with lower physical function after adjustment for prior stroke and other fall risk predictors, indicating that treatment with SSRIs may be associated with lower physical function.

This study has some limitations. Treatment with SSRIs was self-reported and only for the 30 days prior to inclusion, without any data to determine if SSRI use was new or chronic. It should be emphasized that when interpreting these results, we cannot determine if the observed associations were due to extended or short-time SSRI use. Thus, with the available data, we can only report of associations between current SSRI use, physical function and bone traits, without knowledge of how SSRI treatment duration would affect these herein reported associations. In addition, the higher tendency of prior falls in the SSRI-treated group was based on retrospectively data, which is known to be unreliable. Also, this study only included older women within a relative narrow age span and the results cannot be extrapolated to other populations. Furthermore, we were only able to adjust for mental health obtained from the SF-12 questionnaire, and not for a registered diagnosis of depression. This study also has strengths. It is a large population-based study with high-quality data of bone characteristics and well performed and validated clinical tests of physical function.

We conclude that the use of SSRIs was not associated with lower BMD or impaired bone microstructure or geometry, but with poorer physical function, indicating that the previously observed increased fracture risk with SSRI treatment is due to impaired physical function and fall risk, rather than bone fragility.

## Electronic supplementary material

Below is the link to the electronic supplementary material.

Supplementary material 1 (DOCX 17 KB)
