# Supplementary material for: Normal Bone Microstructure and Density But Worse Physical Function in Older Women Treated with Selective Serotonin Reuptake Inhibitors, a Cross-Sectional Population-Based Study
Source: Calcif Tissue Int. 2018 May 5;103(3):278–88. doi: 10.1007/s00223-018-0427-z (PMC6105159; doi:10.1007/s00223-018-0427-z)
Supplement: Supplementary file 1 — Supplementary material 1 (DOCX 17 KB) [file 223_2018_427_MOESM1_ESM.docx]

**Table S1** Number and cause of missing observations

|  | **Main analysis** | | **Subanalysis** | | |  |  |
| --- | --- | --- | --- | --- | --- | --- | --- |
|  | **SSRI No**  (n=971) | **SSRI Yes**  (n=86) | **Matched controls**  (n=344) | **SSRI-treated**  (n=86) | **Reason for missing** | |  |
| *Characteristics* |  |  |  |  |  | |  |
| Appendicular lean mass index (kg/m^2^) | 969 (99.8%) |  |  |  | No DXA scan=2 | |  |
| Fat mass, kg | 969 (99.8%) |  |  |  | No DXA scan=2 | |  |
| Age at menopause, years | 921 (94.8%) | 83 (96.5%) | 327 (95.1%) | 83 (96.5%) | Cannot remember=50, No information=3 | |  |
| Physical activity score (PASE) | 966 (99.5%) |  | 343 (99.7%) |  | Missing component=5 | |  |
| Calcium intake, mg/day | 967 (99.6%) |  | 342 (99.4%) |  | Missing component=4 | |  |
| MCS | 968 (99.7%) |  | 343 (99.7%) |  | Missing component=3 | |  |
| PCS | 968 (99.7%) |  | 343 (99.7%) |  | Missing component=3 | |  |
| Hyperthyreodism, % (n) | 967 (99.6%) |  | 343 (99.7%) |  | No information=4 | |  |
| Hypothyreodism, % (n) | 965 (99.4%) |  | 342 (99.4%) |  | No information=6 | |  |
| Diabetes, % (n) | 970 (99.8%) |  |  |  | No information=1 | |  |
| Chronic liver disease, % (n) | 967 (99.6%) | 85 (98.8%) | 342 (99.4%) | 85 (98.8%) | Answer to question lacking=5 | |  |
| Celiac disease, %, (n) | 967 (99.6%) | 85 (98.8%) | 342 (99.4%) | 85 (98.8%) | Answer to question lacking=5 | |  |
| *Physical function* |  |  |  |  |  | |  |
| One leg standing, s | 737 (75.9%) | 61 (70.9%) | 250 (72.7%) | 61 (70.9%) | Could not perform the test on one or both legs=259 | |  |
| Timed up and go, s | 966 (99.5%) | 85 (98.8%) | 343 (99.7%) | 85 (98.8%) | Could not perform the test=6 | |  |
| Walking speed, m/s | 963 (99.2%) |  | 342 (99.4%) |  | Difficulties to walk 10m=5, No time taken=2, No distance marks in the ground=1 | |  |
| Chair stand test, number/30 s | 910 (93.7%) | 78 (90.6%) | 318 (92.4%) | 78 (90.6%) | Did not want to perform the test=13, Could only stand up with the help from  armrests=54, Could not stand up=2 | |  |
| Grip strength, kg | 926 (95.4%) | 82 (95.3%) | 330 (95.9%) | 82 (95.3%) | Pain=11, Arthritis=13, Rheumatoid arthritis=3, Fracture/operation=8, Paralyzed=4, No fingers=1, Edema=1, Not possible=1 | |  |
| *Bone mineral density* |  |  |  |  |  | |  |
| Lumbar spine | 966 (99.6%) | 85 (98.8%) | 341 (99.1%) |  | Images excluded due to Osteosynthesis material=5, L4 Not included in the analysis=1 | |  |

Included number of women with corresponding percentage is presented for each variable where complete information was absent.
